# Knowledge and Use of Electronic Cigarettes in Young Adults in the United Arab Emirates, Particularly during the COVID-19 Pandemic

**DOI:** 10.3390/ijerph19137828

**Published:** 2022-06-26

**Authors:** Yasir Abbasi, Marie-Claire Van Hout, Mohamed Faragalla, Lynn Itani

**Affiliations:** 1 Maudsley Health, Dubai 1853, United Arab Emirates; yabbasi@nhs.net; 2 International Public Health Policy and Practice, Research and Knowledge Exchange, Public Health Institute, Faculty of Health, Liverpool John Moores University, Liverpool L2 2QP, UK; m.c.vanhout@ljmu.ac.uk; 3 Al Amal Psychiatric Hospital, Emirates Health Services, Dubai 1853, United Arab Emirates; alamal.research@mohap.gov.ae

**Keywords:** smoking, e-cigarettes, COVID-19

## Abstract

(1) Background: The popularity of electronic cigarettes (e-cigarettes) has recently increased. Although they are less harmful than regular cigarettes, they still cause health consequences and their use for smoking cessation is inconclusive. The objective of this study was to evaluate patterns of use, knowledge about, and attitude towards e-cigarettes among youth in the United Arab Emirates (UAE) while also researching the impact of the COVID-19 pandemic on smoking behavior. (2) Methods: An online cross-sectional survey was distributed across three major universities in the UAE (*n* = 240) between March and November 2021. Descriptive analysis, comparison across gender and nationality groups, and correlates between 30-day e-cigarette use and self-reported increases in nicotine consumption during the pandemic were studied. (3) Results: About 37% of students had used an e-cigarette in their lifetime, and 23% had smoked e-cigarettes in the past month. During the pandemic, 52% of university students self-reported no change in nicotine consumption, while only 17.5% had reported an increase. The current smoking of regular cigarettes, waterpipe, and medwakh increased the odds of having an increase in smoking during the pandemic by 5.3 times. (4) Conclusions: The findings inform about youth behavior and knowledge about vaping in the UAE and could also support the development of awareness interventions.

## 1. Introduction

The nicotine market has been evolving due to a dramatic increase in the popularity of novel smoking devices. Electronic cigarettes (e-cigarettes) are one of these devices that are used to deliver nicotine, chemicals (such as propylene glycol, vegetable glycerin), and other flavors and substances [1]. While e-cigarettes have fewer carcinogens than traditional cigarettes, they still expose users to high levels of other toxins that increase the risk of pulmonary and cardiovascular diseases [2,3,4,5]. Although e-cigarettes are considered less harmful than traditional cigarettes, their use as a smoking cessation tool is inconclusive [2,3,4,5]. Recently, a meta-analysis showed a low level of certainty with regards to the effectiveness of e-cigarettes for smoking cessation in the short-term, and there had not been sufficient evidence to prove the safety and effectiveness of e-cigarettes for smoking cessation in the long-term [6]. It is important to note that e-cigarettes may also be a pre-cursor for the use of regular cigarettes and other types of nicotine-containing products [3]. The fact that e-cigarettes have different flavors has also shown to be an important reason for the initiation of vaping among young adults [7]. Given this information, the “Theory of Planned Behavior” (TPB) in health psychology states that whether an individual engages in a health behavior partly depends on his/her attitude towards it [8]. Previous research has also shown that one’s beliefs towards e-cigarettes are a potential risk factor for engagement in the behavior [8].

In addition, at the start of 2020, the world was afflicted by the COVID-19 pandemic that had a global impact not only on public health but also on economies and on the lifestyles of millions. Across different countries, studies have already shown the psychological impact of the pandemic [9,10,11,12]. Use of tobacco and nicotine-containing products, including e-cigarettes, have been associated with a higher risk of COVID-19 complications since they can weaken the immune and respiratory systems [13,14,15,16]. Given the “Theory of Planned Behavior”, it is also important to evaluate whether certain perceptions may have been correlated with self-reported increases in nicotine-consumption during the pandemic [8].

In the United Arab Emirates (UAE), the National Health Survey (2017–2018) was previously conducted by the Ministry of Health and Prevention (MOHAP) and reported that 9.1% of adults currently smoke (15.7% of males, 2.4% of females) and that the average age of smoking commencement was 20.2 years. Among smokers of the age group 18–27, 43.6% were smoking e-cigarettes while 14.5% smoked regular cigarettes, 12.6% smoked water pipes, and 57.3% smoked medwakh [17]. Another recent cross-sectional study across three public universities in the UAE (*n* = 918, average age = 20.7) found that the prevalence of tobacco smoking among university students was 15.1% and that the most common patterns of tobacco use included medwakh (7.2%), regular cigarettes (5.5%), and waterpipe (shisha) (5.5%) [18]. A medwakh, not commonly used in Western countries, is used to smoke a mix (called “dokha”) of dried tobacco with other herbs and spices and has several health consequences including cardiovascular and respiratory issues [18]. In this same study, the prevalence of e-cigarette use was 3.7% and the main reasons for e-cigarette use were: liking the flavors (36.1%), to help quit smoking (34.4%), to have less harm than traditional tobacco for the smokers themselves (24.6%), and for other people (21.3%) [18]. In the study, male students were more likely to be e-cigarette smokers [18]. With regards to the COVID-19 pandemic and effect on smoking, there is one study that looked at lifestyle changes among adults in general in the UAE (*n* = 2060) [19]. The results of that study showed that 21.0% of the respondents had reported an increase in smoking, while there was a decrease for 39.1%, and no change for 39.9%. Females and older adults (>40 years vs. 18–30) were less likely to increase smoking behavior during lockdown [19]. To our knowledge, there has yet been no study in the UAE that evaluates changes in nicotine consumption amidst the pandemic among university students.

Internationally, there have been a few studies on university students that have investigated the change in smoking behavior during the pandemic. A PubMed search with key terms “university”, “tobacco”, and “COVID” showed evidence of more studies showing a decrease in smoking [20,21,22,23] rather than no change [24,25,26] or an increase [27,28].

Due to the surge of novel smoking products among youth and the possible impact of this on future generations, this proposed study aims to evaluate patterns of use, knowledge about, and attitude towards e-cigarettes among youth in the UAE, while also researching the effect of the COVID-19 pandemic on nicotine consumption. This research also incorporates differences across gender and nationality groups (Emirati versus expatriates) with regards to these behaviors. We studied differences between nationals and expatriates due to the unique nature of the UAE population where nationals are only around 12% of the population [29]. The non-Emirati residents (being from around 200 countries) are different to Emirati nationals in terms of culture, traditions, lifestyle, and socio-economic characteristics. The ultimate purpose of this study is to highlight findings that could support the development of interventions that raise awareness about nicotine among university students in the UAE and inform policy-makers about the public health attitudes toward smoking and vaping.

## 2. Materials and Methods

### 2.1. Study Design

This is a cross-sectional survey where data was collected from three major universities in the UAE. In 2022, the population of the UAE was about 10 million, with expatriates representing most of the population [29].

An online survey was sent to all students currently enrolled in the universities regardless of faculty or level (undergraduate, graduate, post-graduate) via email. According to a recent study, the higher education sector in the UAE is dominated by 12 major universities, with three of them being in the public sector and accounting for around 25% of students [30]. Our study included three universities (one private university and two public universities) of these 12 major universities that represent an estimate of around 25% of all university student enrollments in the UAE [30]. This 25% is equivalent to around 33,500 students, so the response rate for our study was 0.7% (*n* = 240). Since UAE citizens can attend university free of charge, around 90% of Emirati students apply for higher education during their last year in secondary school, indicating that UAE citizens have a high university enrollment rate [31]. Data was collected using an online questionnaire with the ISO-certified tool (ISO27001) called Online Surveys (https://www.onlinesurveys.ac.uk/ accessed on 15 February 2020). A total of 240 responses were received.

Data collection initiatives had taken place between the months of March and November 2021. However, all respondents had answered the survey by the end of June 2021. This is because after June 2021 there were summer break months followed by a lack of participation in the study for the 2021–2022 academic year; all students who had been interested had already been recruited. During this time period (March to June 2021), UAE residents received their first cycle of Sinopharm vaccination. It was not until the 2021–2022 academic year that educational institutions started in-person classes again.

### 2.2. Measures

The survey was adapted from questions about e-cigarettes in the Center for Disease Control and Prevention (CDC) National Tobacco Surveys, and also according to the constructs mentioned in this review of survey questions about e-cigarette use [32]. The survey sections included: (i) socio demographics, (ii) use of tobacco products other than e-cigarettes (regular cigarettes, water pipes, and medwakh), (iii) e-cigarette use, (iv) knowledge and perceptions related to e-cigarettes, and (v) the COVID-19 impact on self-reported increases in the consumption of nicotine products.

The lifetime use of e-cigarettes was evaluated with the question: “Have you ever used an electronic cigarette, even just one time in your entire life?” (Yes/No). The 30-day use of e-cigarettes was evaluated with: “During the past 30 days, on how many days did you use e-cigarettes?” The change in nicotine consumption was evaluated with the following question: ”Since the start of COVID-19 in the region (February 2020), how would you describe your use of tobacco products such as regular cigarettes, water pipes, medwakh, and electronic cigarettes?” (Responses: Increased, Decreased, No Change, Don’t Know).

All respondents received a consent form to be reviewed and signed before completing the questionnaire. Prior to data collection, ethical approval was acquired from the ethical review board at the Ministry of Health and Prevention, UAE. Anonymity and confidentiality were preserved throughout the study.

### 2.3. Data Analysis

First, descriptive analysis was conducted for the items in the questionnaire. Chi-square tests were then run to compare patterns of using e-cigarettes and knowledge about e-cigarettes across gender and nationality groups (Emirati vs. other nationalities).

As a next step, the following primary outcomes were set as dependent variables: “30-day use of e-cigarettes” and “increase in nicotine-product consumption during the pandemic”. Simple logistic regression was first run with several covariates followed by multiple logistic regression models that included only covariates with *p*-value less than 0.2 in the simple logistic regression. Unadjusted and adjusted odds ratios (OR’s) were reported along with 95% confidence intervals. A *p*-value less than 0.05 was considered to be statistically significant.

The following were considered main predictors for the simple and the multiple logistic regression models: current smoking of tobacco products (regular cigarettes, waterpipe, and medwakh) (Yes/No); having a parent who currently smokes regular cigarettes, waterpipe, and medwakh (Yes/No); having a close peer who currently smokes regular cigarettes, waterpipe, and medwakh (Yes/No); thinking that e-cigarettes have moderate/high levels of harm to health (Yes/No); believing that e-cigarettes are addictive (Yes/No); having received information about e-cigarettes through a media source (Yes/No) (the following media sources were considered: social media like Facebook or Twitter; online advertising; television advertisement; radio advertisement; billboards and/or public signs; newspapers or magazines); and thinking that tobacco increases the risk of COVID-19 and its complications (Yes/No). Media information had been studied since in the literature, there is evidence that increased exposure to e-cigarette advertisements is associated with higher likelihood of e-cigarette use [33]. Other covariates adjusted for in the regression models included socio-demographics: female gender, age (numerically), and Emirati nationality (Yes/No). Data analysis was conducted using Statistical Package for Social Sciences (SPSS).

## 3. Results

### 3.1. Socio-Demographic Characteristics and Current Use of Regular Cigarettes, Waterpipe or Medwakh

The sample was comprised of 75.4% females and was also 70.0% Emirati nationality. The study participants were mostly residents of Abu Dhabi (58.3%) and Dubai (18.8%). Most were between the age of 18 and 24 (91.3%). The majority of respondents had high-school for their highest level of education (64.6%) and had a single marital status (94.6%).

The following was the description of current smoking for products other than e-cigarettes: 6.7% smoke regular cigarettes, 12.9% smoke waterpipe, and 10.0% smoke medwakh. In addition, 20.4% currently smoke any of these tobacco products. With regards to their social network, 27.1% had at least one parent that currently and regularly smokes cigarettes, waterpipe, or medwakh, and 44.6% had a peer that currently smokes any of these products. Further details about the characteristics of the sample are presented in Table 1.

### 3.2. Patterns of Use and Knowledge about E-Cigarettes

A total of 90.4% of the sample had previously heard of e-cigarettes, however only about a third (36.7%) of the sample had ever used an e-cigarette device and about one-fifth (22.5%) of the sample had used an e-cigarette device in the past month. Among those who had used e-cigarettes at least once in their life, the following was the frequency in the past month: not at all (38.6%), rarely (20.5%), some days (17.0%), and every day (23.9%); 27.3% had tried to stop using e-cigarettes at some point, while 53.4% believed that this question was not applicable due to their rare use of the device. In the past 12 months, only 7.1% of those who had used an e-cigarette tried to completely switch from smoking regular cigarettes to using e-cigarettes. The top five reasons for having ever used e-cigarettes were the following: friend or family member used them (64.8%), to try to quit using other tobacco products such as regular cigarettes (27.3%), availability in flavors such as mint, candy, fruit, or chocolate (19.6%), less harmful than other forms of tobacco such as regular cigarettes (15.0%), and can be used in areas where other tobacco products such as regular cigarettes are not allowed (9.2%). The main sources of information about e-cigarettes were social media such as Facebook, Instagram, or Twitter (67.1%), online advertising (29.2%), and newspapers or magazines (12.9%). Table 2 shows further information about knowledge concerning e-cigarettes and patterns of use of e-cigarettes, along with comparisons across gender and nationality.

### 3.3. Studying the COVID-19 Impact on Nicotine Consumption

Since the start of the COVID-19 pandemic, around half of the university students confirmed that they had observed no change in their self-reported consumption of nicotine products (regular cigarettes, e-cigarettes, waterpipe, and medwakh) and also estimated the same for their parents (Table 2). Differences across gender and nationality with regards to nicotine consumption since the start of the pandemic are also shown in Table 2. In the sample, 67.5% believed that smoking increases the risk of COVID-19 and its complications while 7.5% did not believe that smoking increases the risk; 25.0% did not know. Of those who have used e-cigarettes at least once before, 62.5% had knowledge that their use increases the risk of COVID-19 while 21.6% had no knowledge about this topic and 15.9% believed there was no increase in risk. During the pandemic, most respondents (64.6%) had not used an e-cigarette device (and 6.3% responded did not know). Only a minority (13.8%) had shared an e-cigarette device with others, while 15.4% had used an e-cigarette device yet not shared it with others.

### 3.4. Correlates of 30-Day E-Cigarette Use and an Increase in Self-Reported Nicotine Product Consumption since the Start of the Pandemic

For correlates of 30-day e-cigarette use, the results of simple logistic regression are shown in Table 3. The following variables were still significant in the multi-logistic model: current smoking of other tobacco products such as regular cigarettes, waterpipe, and medwakh (OR = 12.887, CI = 5.255–31.600, *p* < 0.001) and believing that e-cigarettes have a moderate or high level of harm to a person’s health (OR = 0.150, CI = 0.030–0.738, *p* = 0.020).

For the correlates of an increase in smoking since the start of the pandemic, the results of the simple logistic regression are also shown in Table 3. The following variable was still significant in the multi-logistic regression model: current smoking of regular cigarettes, waterpipe, or medwakh (OR = 5.310, CI = 2.224–12.675, *p* < 0.001).

## 4. Discussion

The results of this study show that around one-third of the respondents had used an e-cigarette device at least once before, and about one-fifth had used e-cigarettes in the past month. In previous research, 3.7% of university students in the UAE were current e-cigarette smokers [18]. For other countries in the Gulf Corporation Council (GCC) region, the prevalence of e-cigarette use among university students had been estimated at 14% when data were collected in 2020 within Qatar [34], and 7.2% and 10.6% among university students in Saudi Arabia within a study published in 2020 and another conducted in 2018, respectively [35,36].

With regards to changes in the consumption of nicotine products since the start of the pandemic, around half of the university students (52.1%) affirmed to no change in consumption and there was an increase in only 17.5% of respondents, a decrease for 6.7%, and a response of “don’t know” for 23.8%. In the multi-logistic regression model, an increase in consumption during the pandemic was also significantly associated with current smoking of nicotine products other than e-cigarettes. Previous research about changes in smoking among the entire adult population in the UAE during the pandemic had shown comparable levels of increase and no change (21% and 39.9%, respectively), but a different level of decrease (39.1%) [19].

With regards to the logistic regression models, we had looked at correlates of 30-day e-cigarette smoking in addition to correlates of self-reported increase in nicotine product consumption during the pandemic. After adjusting for other variables, smoking e-cigarettes in the past 30 days was correlated with current smoking of other types of tobacco products (regular cigarettes, waterpipe, and medwakh), and the belief that e-cigarettes have moderate to high levels of harm to a person′s health had a protective effect. This is consistent with previous literature where smokers of conventional cigarettes were more likely to also use e-cigarettes [37,38]. Other cross-sectional research studies have also shown a correlation between e-cigarette use and lower perceived harm [39,40]. Being a current smoker of regular cigarettes, waterpipe, or medwakh also had a 5.3 times increase in odds for reporting an increase in nicotine product consumption during the pandemic. Being female was also borderline protective for such an increase [19].

For differences across gender, we found that males have significantly higher levels of lifetime use of e-cigarette devices (61.0% vs. 28.7%) and 30-day use of e-cigarette devices (40.7% vs. 16.6%). Males also have a significantly lower perception of the level of harm of e-cigarettes to a person’s health; this is evident since 33.9% believe that e-cigarettes are “very harmful” to a person’s health (compared to 60.2% of females). Additionally, 55.9% believe that e-cigarettes are addictive (compared to 78.5% of females). Lastly, 45.8% believe that e-cigarettes are a helpful aid for smoking cessation (compared to 23.2% of females). This shows that young men are more likely to use e-cigarettes in the UAE and to perceive them as less harmful to one’s health. This is consistent with previous research about tobacco smoking among university students in the UAE, where males are more likely to consume tobacco [18]. In the GCC, this pattern has also been observed in Bahrain, Saudi Arabia, and Yemen [41,42,43,44,45]. This could be due to smoking being more socially acceptable for males in this region, and that they could also be subject to peer influence [42].

For differences across nationality groups, we found no significant differences with regards to lifetime and 30-day use of e-cigarettes, nor with knowledge and attitudes towards e-cigarettes (Table 2). Emirati nationality had also not been significantly correlated with a self-reported increase in nicotine consumption during the pandemic (Table 3).

Among those who had ever used e-cigarettes, we found that the most common reason for this usage was having a friend or family member who had used them. Additionally, information about these devices had mostly been received through social media networks. It is important to note that e-cigarettes are often advertised by merchants and manufacturers as a smoking cessation tool, and they could have recently gained popularity due to their recent legalization in the country [46]. However, the use of these devices is still a threat to public health [47]. In our study, we found that around a third of respondents in the total sample have the perception that these devices are helpful for smoking cessation. In addition, 27.3% of those who had ever used e-cigarettes had done so to help them quit using other tobacco products such as regular cigarettes. This is a concern, since these e-cigarettes are also addictive (due to nicotine dependence) and around 70% of the sample was aware of that.

Our research found that around 27.3% of respondents had at some point tried to stop using e-cigarettes. This could be due to several interventions that the UAE has recently taken with the goal of reducing nicotine use in the country; these measures include imposing a one-hundred percent “excise tax” on nicotine products, the prohibition of smoking in public places (including educational institutions), controls on advertising in the media, and the implementation of specialized MOHAP smoking cessations clinics [46].

Despite these significant findings, the study has several limitations. First, the use of a convenience sample limits the response rate and the sample size, thus influencing the generalizability of the results and the potential under-representation or over-representation of certain groups. Regardless of that, we believe that the study still provides useful information about nicotine consumption and e-cigarette use in the region while considering the limitations of a convenience sample. We found that the majority of respondents were female, hence showing a potential bias from gender skew. It is also to be noted that the study is about youth in the UAE, and yet the survey was only distributed to university students; there is also a youth segment of the labor workforce in the country. The survey was also based on self-reporting, and this may have created a social desirability reporting bias (due to cultural reasons), especially among females. Given the self-reporting nature of the evaluation of change in behavior during the pandemic, retrospective reports of COVID-related smoking behavior change may not necessarily align with longitudinal assessments of change [48]. The questionnaire did not evaluate the use of other electronic nicotine products such as e-shisha/e-waterpipe and e-pipes/e-cigars. Although there has been evidence of use of these devices in the region, we did not investigate if having a family or friend who is specifically an e-cigarette smoker is correlated with a user being an e-cigarette smoker themselves [36,49]. Lastly, the study considered students to be informants on behalf of their parents, and so the responses about parents may not have been accurate. Despite these limitations, this is the first study to investigate nicotine consumption patterns among university students during the pandemic in the UAE. This study also provides baseline information about youth in the country with the hope to also encourage further research about the topic in the UAE and neighboring countries.

## 5. Conclusions

In conclusion, this study has provided data about patterns of e-cigarette use, as well as knowledge and attitudes towards e-cigarette devices for youth in the region. Current smoking of other tobacco products (regular cigarettes, waterpipe, and medwakh) was highly correlated with e-cigarette use in the past 30 days. The results show that the most common reason for youth to have initiated vaping was having a friend or a family member who smokes e-cigarettes. Information about these devices had mostly been received through social media networks. The findings show that only a minority of university students had self-reported an increase in their consumption of nicotine products during the pandemic. The results support the development of various public health prevention initiatives such as increasing awareness through circulars, university policies, social media, and other educational campaigns that also specifically target males since they seem to have an overall lower perceived harm of e-cigarette smoking.

## Figures and Tables

**Table 1:** Socio-demographic characteristics and current use of regular cigarettes, waterpipe, or medwakh (*n* = 240).

	N	%
**Gender**		
Male	59	24.6
Female	181	75.4
**Age**		
18 to 24	219	91.3
25 and above	21	8.8
**Nationality**		
Emirati	168	70.0
Other nationality	72	30.0
**Emirate of residence**		
Abu Dhabi	140	58.3
Dubai	45	18.8
Northern Emirates	55	22.9
**Highest degree**		
Highschool	155	64.6
Bachelor	75	31.3
Masters	8	3.3
Others	2	0.8
**Marital status**		
Single	227	94.6
Married	10	4.2
Divorced	1	0.4
Widowed	2	0.8
**Currently smokes regular cigarettes**	16	6.7
**Currently smokes waterpipe**	31	12.9
**Currently smokes medwakh**	24	10.0
**Currently smokes any of regular cigarettes, waterpipe, or medwakh**	49	20.4
**Any parent currently smokes regular cigarettes, waterpipe, or medwakh**	65	27.1
**Any close peer currently smokes regular cigarettes, waterpipe, or medwakh**	107	44.6

**Table 2:** Patterns of use and knowledge about e-cigarettes across gender and nationality.

	% Among Males (*n* = 59)	% Among Females (*n* = 181)	χ2	*p*-Value	% Among Other Nationalities (*n* = 72)	% Among Emiratis (*n* = 168)	χ2	*p*-Value	Total (*n* = 240)
**Use of e-cigarettes**									
Have ever used an e-cigarette before	61.0	28.7	19.975	<0.001	34.7	37.5	0.167	0.682	36.7
30-day use of e-cigarette	40.7	16.6	14.825	<0.001	18.1	24.4	1.165	0.280	22.5
**Knowledge and attitudes towards e-cigarettes**									
*How harmful do you think using electronic cigarettes are to a person’s health?*									
Not harmful at all	8.5	3.9	12.712	0.002	5.6	4.8	2.614	0.271	5.0
Moderately harmful	57.6	35.9			48.6	38.1			41.3
Very harmful	33.9	60.2			45.8	57.1			53.8
*Do you think that electronic cigarettes could be less harmful than regular cigarettes?*									
Could be less harmful	50.8	35.4	6.345	0.042	45.8	36.3	3.551	0.169	39.2
All equally harmful	28.8	47.0			33.3	46.4			42.5
Don′t know	20.3	17.7			20.8	17.3			18.3
*Do you believe that electronic cigarettes are addictive?*									
No	22.0	5.5	16.668	<0.001	15.3	7.1	4.526	0.104	9.6
Yes	55.9	78.5			65.3	76.2			72.9
Don′t know	22.0	16.0			19.4	16.7			17.5
*Do you believe electronic cigarettes are a helpful aid for smoking cessation?*									
No	30.5	48.6	11.505	0.003	41.7	45.2	2.982	0.225	44.2
Yes	45.8	23.2			36.1	25.6			28.7
Don′t know	23.7	28.2			22.2	29.2			27.1
**Self-reported changes in consumption of nicotine-containing products (regular cigarettes, e-cigarettes,** **waterpipe, and medwakh) since the start of the pandemic**									
*Self-evaluation of change in consumption*									
Decreased	6.8	6.6	13.645	0.003	6.9	6.5	9.720	0.021	6.7
No change	52.5	51.9			66.7	45.8			52.1
Increased	30.5	13.3			11.1	20.2			17.5
Don’t know	10.2	28.2			15.3	27.4			23.8
*Students’ evaluation of parents’ change in consumption*									
Decreased	10.2	3.9	5.823	0.121	6.9	4.8	7.345	0.062 *	5.4
No change	57.6	50.8			62.5	48.2			52.5
Increased	8.5	15.5			13.9	13.7			13.8
Don’t know	23.7	29.8			16.7	33.3			28.3

**Table 3:** Correlates of 30-day e-cigarette use and increase in consumption of nicotine products during the pandemic.

Model 1	30-Day E-Cigarette Use (*n* = 54)							
	Simple Logistic Regression (Bivariate Regression)				Multiple Logistic Regression (Multi-Variate Regression)			
	OR	CI		*p*-Value	OR	CI		*p*-Value
Male gender	3.451	1.801	6.615	<0.001	2.013	0.846	4.791	0.114
Age (continuous)	0.956	0.862	1.060	0.392				
Emirati nationality	1.465	0.730	2.939	0.282				
Currently smokes tobacco *	22.632	10.373	49.378	<0.001	12.887	5.255	31.600	<0.001
Any parent currently smokes tobacco *	0.353	0.187	0.670	0.001	0.652	0.278	1.528	0.325
Any close peer currently smokes tobacco *	0.197	0.100	0.388	<0.001	0.480	0.203	1.138	0.096
Thinks that e-cigs have moderate/high levels of harm to health	0.126	0.036	0.438	0.001	0.150	0.030	0.738	0.020
Believes that e-cigs are addictive	0.757	0.391	1.467	0.410				
Has received info through media about e-cigs	1.816	0.875	3.771	0.110	2.122	0.821	5.483	0.120
Thinks that tobacco increases risk of COVID-19 and its complications	0.353	0.182	0.684	0.002	0.589	0.250	1.389	0.227
**Model 2**	**Increase in self-reported nicotine product consumption** **during pandemic (*n* = 42)**							
	**Simple Logistic Regression** **(bivariate regression)**				**Multiple Logistic Regression** **(multi-variate regression)**			
	**OR**	**CI**		***p*-value**	**OR**	**CI**		***p*-value**
Male gender	2.872	1.425	5.790	0.003	2.174	0.969	4.875	0.060
Age (continuous)	0.953	0.848	1.071	0.421				
Emirati nationality	2.030	0.889	4.635	0.093	2.355	0.952	5.827	0.064
Currently smokes tobacco *	6.967	3.361	14.442	<0.001	5.310	2.224	12.675	<0.001
Any parent currently smokes tobacco *	0.795	0.384	1.643	0.535				
Any close peer currently smokes tobacco *	0.426	0.215	0.844	0.015	0.911	0.398	2.082	0.825
Thinks that e-cigs have moderate/high levels of harm to health	1.064	0.224	5.043	0.938				
Believes that e-cigs are addictive	1.231	0.567	2.672	0.600				
Has received info through media about e-cigs	1.633	0.736	3.620	0.228				
Thinks that tobacco increases risk of COVID-19 and its complications	0.578	0.290	1.153	0.120	0.868	0.400	1.884	0.720

*: tobacco product such as regular cigarettes, waterpipe, and medwakh.

## Data Availability

The data presented in this study is available upon request from the corresponding author.
